# Analysis of Neurological Adverse Events Reported in VigiBase From COVID-19 Vaccines

**DOI:** 10.7759/cureus.21376

**Published:** 2022-01-18

**Authors:** Siddhartha Dutta, Rimplejeet Kaur, Jaykaran Charan, Pankaj Bhardwaj, Sneha R Ambwani, Shoban Babu, Jagdish P Goyal, Mainul Haque

**Affiliations:** 1 Department of Pharmacology, All India Institute of Medical Sciences, Rajkot, Rajkot, IND; 2 Department of Pharmacology, All India Institute of Medical Sciences, Jodhpur, Jodhpur, IND; 3 Department of Community Medicine & Family Medicine and School of Public Health, All India Institute of Medical Sciences, Jodhpur, Jodhpur, IND; 4 Department of Pediatrics, All India Institute of Medical Sciences, Jodhpur, Jodhpur, IND; 5 Department of Pharmacology and Therapeutics, National Defence University of Malaysia, Kuala Lumpur, MYS

**Keywords:** sars-cov-2 (severe acute respiratory syndrome coronavirus -2), adverse events following immunization, azd1222, bnt162b2, mrna-1273, neurological disorders, covid-19 vaccines, adverse events

## Abstract

Background: Fifteen COVID-19 vaccines have been granted emergency approval before the completion of conventional phases of clinical trials. The present study aimed to analyze the neurological adverse events (AEs) post-COVID-19 vaccination and focuses on determining the association of AEs with the vaccine.

Methodology: The neurological AEs reported for COVID-19 vaccines in the WHO pharmacovigilance database (VigiBase) were extracted from the System Organ Classes - neurological disorders and investigations. Descriptive statistics are reported as percentage and frequency and the disproportionality analysis was also conducted.

Results: For the neurological system, 19,529 AEs were reported. Of these, 15,638 events were reported from BNT162b2 vaccine, 2,751 from AZD1222 vaccine, 1,075 from mRNA-1273 vaccine, eight from Vero vaccine, two from Covaxin, and for 55 AEs, vaccine name was not mentioned. The reason for more AEs reported with BNT162b2 can be maximum vaccination with BNT162b2 vaccine in the study period. According to the disproportionality analysis based on IC_025_ value, ageusia, anosmia, burning sensation, dizziness, facial paralysis, headache, hypoaesthesia, lethargy, migraine, neuralgia, paresis, parosmia, poor sleep quality, seizure, transient ischemic attack, and tremor are some of the AEs that can be associated with the administration of the vaccine.

Conclusion: The vaccines should be monitored for these AEs till the causality of these AEs with COVID-19 vaccines is established through further long-term follow-up studies. These neurological AEs reported in VigiBase should not be taken as conclusive and mass vaccination should be carried out to control the pandemic until a definite link of these adverse effects is established.

## Introduction

COVID-19 is an acute respiratory illness caused by the SARS-CoV-2 virus. Since its initial report in December 2019 in Wuhan, China, COVID-19 has rattled the global research to combat this deadly virus. As of now on April 7, 2021, it has infected 270,791,973 individuals including 5,318,216 deaths [1]. The sudden spike in the number of cases led to a shortage of various medicines and personal protective equipment in many countries and imposed a lockdown to stop the spread of the virus further worsening the daily life of people across the globe [2-4]. The research related to the COVID-19 virus is exploring mainly four arenas- dissecting the virus itself, exploring the diagnostic tools, finding the prevention and the treatment modalities. Various modalities, repurposed drugs, complementary and alternative medicines, vitamins, nutraceuticals, and immune-boosters were being used to tackle the condition as there was no definitive treatment [5-9]. The drugs being explored for treatment include drugs like hydroxychloroquine, remdesivir, favipiravir, tocilizumab, ivermectin, baricitinib, etc. Numerous studies were conducted to observe the safety and efficacy of these drugs in COVID-19 [6,7,10-12]. As of May 19, 2021, 15 vaccines were approved for COVID-19 namely Comirnaty (BNT162b2), Moderna COVID‑19 vaccine (mRNA-1273), AstraZeneca COVID-19 vaccine (AZD1222); also known as Vaxzevria and Covishield, Sputnik V, Sputnik Light, COVID-19 vaccine Janssen (JNJ-78436735; Ad26.COV2.S), CoronaVac, BBIBP-CorV, EpiVacCorona, Convidicea (Ad5-nCoV), Covaxin, WIBP-CorV, CoviVac, ZF2001, and QazVac (QazCovid-in) [13]. In view of the initial pandemic scenario, all vaccines were granted emergency approval trials based on the data generated from the initial phases of clinical trials, before completion of all the phases of a clinical trial [14-16]. The remaining phases of clinical trials were continuing to confirm their safety and efficacy. Thus, it was imperative to monitor the adverse events reported post-COVID-19 vaccination. There were scattered reports of neurological adverse events following the COVID-19 vaccination [17-19]. Thus, the present study was planned by the authors in order to evaluate the neurological adverse events reported in the WHO pharmacovigilance database, VigiBase.

VigiBase is the global post-marketing pharmacovigilance database maintained by WHO, which contains the adverse events of approved drugs reported from all over the world [20]. VigiBase database had been used previously to analyze the safety profile of many drugs and vaccines including the therapies used for treatment and prevention of COVID-19 [6,7,10,11,21,22]. We evaluated all the neurological adverse events reported for COVID-19 vaccines in VigiBase. We also analyzed if there exists any relationship between the reported adverse events and the vaccines. This study was also planned to generate a safety signal for COVID-19 vaccines in the prime stage and provide a platform for other studies for generating or detecting the safety data of COVID-19 vaccines.

## Materials and methods

The authors used VigiBase for analysis of adverse events related to COVID-19 Vaccines reported between December 15, 2020, and January 24, 2021. VigiBase is a global pharmacovigilance database established in 1978 and consists of over 20 million reports of suspected adverse events reported since its origin by its 130 member countries which represent 90% of the world population [20,23,24].

VigiBase is linked with MedDRA, WHO-ART, WHO-ICD in order to facilitate uniform data entry, retrieval, and analysis. The adverse events are reported as Individual Case Safety Report (ICSR). ICSR is also termed as “spontaneous” or “voluntary” when the reports are generated in the post-marketing phase of the drug when it is available for general use [20,23-25].

The adverse events reported in VigiBase are in a structured form that consists of information regarding three domains: patient (age, gender, country, and continent of residence), drug (name, start and end date, dose, route of administration, and the indication for use), and adverse event (type of event, onset date, seriousness, causality, and the outcome). The drugs are coded in VigiBase in alliance with the WHO Drug Dictionary enhanced including the Anatomical Therapeutic Classification. The adverse events are also reported and coded in accordance with the Medical Dictionary for Regulatory Authorities (MedDRA) and the WHO adverse reaction Terminology [25,26]. The information in the MedDRA is contained as highly standardized medical terminology to allow the global sharing of consistent regulatory information for drugs used by humans [27,28]. In the VigiBase, the information related to adverse events is recorded in accordance with MedDRA in a highly specific hierarchical order containing five levels: lowest level terms (LTTs), preferred terms (PTs), high-level terms (HLTs), high-level group terms (HLGTs), and system organ classes (SOCs) [28,29].

In the present study, the SOC and PT were used for analysis. The PT contains the specific disease, symptom, therapeutic indication, and surgical or medical procedures. The PT is arranged into SOC according to the etiology (infections and infestations), manifestation site (e.g., neurological disorder, musculoskeletal disorder), or purpose (medical or surgical procedure) [30].

The present study incorporated all suspected neurological adverse events reported in VigiBase after administration of COVID-19 vaccines - BNT162b2, AZD1222, mRNA-1273, Covaxin, and unknown vaccines (vaccine for which no name is mentioned in the ICSR form) between December 15, 2020, and January 24, 2021. The authors extracted SOC - neurological disorders and investigations from the database. The SOC investigation was cleaned further to remove all adverse events except for those related to the neurological system. The individual neurological adverse events are reported as frequency and percentage. The authors also used disproportionality analysis, the method of signal detection for the adverse events that are spontaneously reported in the database. In disproportionality analysis, the Frequentist and the Bayesian information component (IC) methods are applied to compare the drug adverse event pair with the other drug adverse events pairs of the database to evaluate if the observed frequency of the events for a drug is more than expected [31-35].

The IC was used by the authors to evaluate the relationship between the specific adverse events to COVID-19 vaccine administration. IC is a Bayesian method of signal generation, and it can avoid false-positive results when events are low [31,35]. In order to link a particular adverse event to a specific drug (COVID-19 vaccine in the current study), the lower limit of IC_025_ should have a positive value. Since in the present study, the frequency of many adverse events was less than four, thus the authors used only IC_025_ values and not the ROR or PRR values. Although, in this study, the reporting odds ratios (ROR) and proportional odds ratios (POR) were not used to link the events with the vaccines, but for the events with positive IC_025_, the ROR and POR with 95% credibility Interval were also mentioned. The event was not considered to be linked to the vaccine if its IC_025_ value was negative and the ROR or PRR was more than 1. The IC_025_ values were calculated separately for the different age groups and genders. Descriptive statistics were reported in the form of frequency and percentage. Statistical Package for Social Science version 17 (SPSS Inc., Chicago, IL, USA) was used for analysis. Institutional Ethics Committee exempted this project from the ethics review as this study is based on secondary data analysis which involves no direct contact with any human subject.

## Results

This study is based on adverse events reported in the VigiBase database from December 15, 2020 to January 24, 2021. In this period, 103,954 adverse events were reported from 30,532 subjects who were administered the COVID-19 vaccine. Out of 103,954, 19,529 AEs were related to clinical events and investigations related to the neurological system. Total 15,638 events were reported from the BNT162b2 vaccine, 2,751 from AZD1222 vaccine, 1,075 from mRNA-1273 vaccine, eight from Vero vaccine, two from Covaxin, and for 55 AEs vaccine name was not mentioned. Neurological AEs reported from the abovementioned vaccines are summarized in Supplementary Tables 1-6.

As per the disproportionality analysis based on IC025 value, ageusia, allodynia, anesthesia, anosmia, aura, balance disorders, burning sensation, cervicobrachial syndrome, cluster headache, dizziness, postural dizziness, dysgeusia, exertional headache, facial paralysis, facial paresis, facial spasm, febrile convulsion, head discomfort, headache, hemiparaesthesia, hemiparesis, hyperaesthesis, hypersomnia, hypoaesthesia, hypogeusia, hyperresponsive to stimuli, hyposomnia, ischemic stroke, lethargy, loss of consciousness, migraine, migraine with aura, monoparesis, neuralgia, paraesthesia, paresis, parosmia, petit mal epilepsy, poor sleep quality, presyncope, seizure, sensory disturbance, sensory loss, sinus headache, syncope, taste disorder, tension headache, transient global amnesia, transient ischemic attack, tremor, tunnel vision, and unresponsive to stimuli are the AEs, which can be considered to be associated with the administration of the vaccine (Table 1).

**Table 1:** Disproportionality analysis of various neurological adverse events associated with COVID-19 vaccines. OR - odd ratios; POR - proportional odd ratios

TermText	Stratification (Full database/ Age/Gender)	Population	OR	IC025	POR
Ageusia	Age group	18 - 44 years	6.3 (5.0-7.9)	2.223	6.2(5.0-7.8)
Ageusia	Age group	Age group 45 - 64 years	4.1(3.1-5.3)	1.567	4.0(3.1-5.3)
Ageusia	Age group	Age Group unknown	4.9(2.9-8.3)	1.245	4.9(2.9-8.2)
Ageusia	Gender	Gender Male	5.8(4.3-7.9)	1.968	5.8 (4.3-7.8)
Ageusia	Gender	Gender Female	4.0(3.3-4.8)	1.668	3.9(3.3-4.8)
Ageusia	Full database	All subjects	4.4(3.7-5.1)	1.864	4.4(3.7-5.1)
Allodynia	Age group	Age group 18 - 44 years	14.1(6.2-31.9)	1.404	14.1(6.2-31.9)
Allodynia	Gender	Gender Female	8.6(3.6-20.8)	0.808	8.6(3.6-20.8)
Allodynia	Full database	All subjects	11.2(5.3-23.6)	1.466	11.2(5.3-23.6)
Anesthesia	Age group	Age group 45 - 64 years	80.2(38.1-168.8)	2.621	80.2(38.1-168.5)
Anesthesia	Gender	Gender Female	34.6(17.7-68.0)	2.520	34.6(17.7-68.0)
Anesthesia	Full database	All subjects	32.1(17.0-60.5)	2.640	32.1(17.0-60.4)
Anosmia	Age group	Age group 18 - 44 years	10.3(7.9-13.3)	2.808	10.2(7.9-13.3)
Anosmia	Age group	Age group 45 - 64 years	5.9(4.3-8.1)	1.976	5.9(4.3-8.1)
Anosmia	Age group	Age group 65 - 74 years	11.3(4.2-30.3)	0.658	11.3(4.2-30.0)
Anosmia	Age group	Age Group unknown	12.2(7.7-19.9)	2.508	12.1(7.7-18.9)
Anosmia	Gender	Gender Male	12.8(9.3-17.6)	2.959	12.7(9.3-17.5)
Anosmia	Gender	Gender Female	6.3(5.1-7.9)	2.271	6.3(5.1-7.8)
Anosmia	Full database	All subjects	7.7(6.5-9.3)	2.630	7.7(6.5-9.2)
Aura	Age group	Age group 18 - 44 years	5.0(2.2-11.2)	0.548	5.0(2.2-11.2)
Aura	Gender	Gender Female	3.7(1.6-8.2)	0.224	3.7(1.6-8.2)
Aura	Gender	Gender Male	11.2(3.6-34.8)	0.133	11.2(3.6-34.7)
Aura	Full database	All subjects	4.9(2.6-9.5)	0.935	4.9(2.6-9.5)
Balance disorder	Age group	Age group ≥ 75 years	2.2(1.4-3.4)	0.422	2.2(1.4-3.4)
Balance disorder	Gender	Gender Male	1.7(1.1-2.4)	0.128	1.7(1.1-2.4)
Burning sensation	Age group	Age group 18 - 44 years	1.5(1.2-1.8)	0.186	1.5 (1.2-1.8)
Burning sensation	Age group	Age group 45 - 64 years	1.6(1.2-2.1)	0.283	1.6(1.2-2.1)
Burning sensation	Gender	Gender Female	1.4(1.1-1.6)	0.185	1.4(1.1-1.6)
Burning sensation	Full database	All subjects	1.5(1.3-1.8)	0.356	1.5(1.3-1.8 )
Cervicobrachial syndrome	Gender	Gender Female	7.0(2.9-17.0)	0.645	7.0(2.9-17.0)
Cervicobrachial syndrome	Full database	All subjects	7.4(3.3-16.6)	0.929	7.4(3.3-16.6)
Cluster headache	Age group	Age group 18 - 44 years	15.2(9.2-25.1)	2.566	15.1(9.2-25.0)
Cluster headache	Age group	Age group 45 - 64 years	18.7(10.5-33.2)	2.493	18.6(10.5-33.1)
Cluster headache	Gender	Gender Female	24.0(16.4-35.0)	3.468	24.0(16.4-35.0)
Cluster headache	Full database	All subjects	19.5(13.6-27.9)	3.333	19.5(13.6-27.8)
Dizziness	Age group	Age group 0 - 27 days	15.3(5.4-43.6)	0.761	13.6(5.4-34.4)
Dizziness	Age group	Age group 28 days to 23 months	81.3(46.6-141.8)	3.530	73.3(44.4-121.2)
Dizziness	Age group	Age group 2 - 11 years	4.7(2.3-9.7)	0.723	4.5(2.3-8.7)
Dizziness	Age group	Age group 18 - 44 years	2.6(2.5-2.7)	1.206	2.4(2.3-2.5)
Dizziness	Age group	Age group 45 - 64 years	2.2(2.1-2.4)	0.973	2.1(2.0-2.2)
Dizziness	Age group	Age group 65 - 74 years	2.2(1.7-2.8)	0.679	2.1(1.7-2.6)
Dizziness	Age group	Age group ≥ 75 years	1.4(1.2-1.6)	0.191	1.4(1.2-1.6)
Dizziness	Age group	Age Group unknown	3.1(2.7-3.6)	1.334	3.0(2.6-3.4)
Dizziness	Gender	Gender Male	2.2(2.0-2.5)	0.971	2.2(2.0-2.4)
Dizziness	Gender	Gender Female	2.7(2.6-2.8)	1.279	2.5(2.4-2.6)
Dizziness	Gender	Gender not known	4.4(3.4-5.7)	1.630	4.2(3.3-5.3)
Dizziness	Full database	All subjects	2.8(2.7-2.9)	1.356	2.7(2.6-2.7)
Dizziness postural	Age group	Age group 18 - 44 years	21.1(16.7-26.6)	3.790	21.0(16.7-26.4)
Dizziness postural	Age group	Age group 45 - 64 years	13.7(10.2-18.5)	3.095	13.7(10.2-18.5)
Dizziness postural	Age group	Age group 65 - 74 years	16.4(6.8-39.6)	1.239	16.3(6.8-39.1)
Dizziness postural	Age group	Age group ≥ 75 years	6.8(3.2-14.4)	1.030	6.8(3.2-14.3)
Dizziness postural	Age group	Age Group unknown	13.8(6.9-27.7)	1.802	13.7(6.9-27.5)
Dizziness postural	Gender	Gender Male	8.5(5.3-13.5)	2.065	8.4(5.3-13.4)
Dizziness postural	Gender	Gender Female	17.4(14.5-20.9)	3.711	17.3(14.5-20.8)
Dizziness postural	Gender	Gender not known	48.6(19.9-118.5)	1.654	48.3(19.9-117.2)
Dizziness postural	Full database	All Subjects	15.5(13.1-18.3)	3.605	15.4(13.1-18.2)
Dysgeusia	Age group	Age group 18 - 44 years	3.7(3.3-4.3)	1.665	3.7(3.2-4.2)
Dysgeusia	Age group	Age group 45 - 64 years	3.7(3.2-4.3)	1.642	3.7(3.2-4.2)
Dysgeusia	Age group	Age group 65 - 74 years	3.1(1.7-5.8)	0.458	3.1(1.7-5.7)
Dysgeusia	Age group	Age Group unknown	3.1(2.2-4.5)	0.998	3.1(2.1-4.5)
Dysgeusia	Gender	Gender Male	2.6(2.0-3.4)	0.950	2.6(2.0-3.4)
Dysgeusia	Gender	Gender Female	3.6(3.3-4.0)	1.683	3.6(3.2-4.0)
Dysgeusia	Gender	Gender not known	7.6(4.9-11.9)	1.982	7.5(4.8-11.5)
Dysgeusia	Full database	All Subjects	3.7(3.4-4.1)	1.733	3.7(3.4-4.0)
Dysstasia	Age group	Age group 18 - 44 years	2.5(1.6-4.0)	0.557	2.5(1.6-4.0)
Exertional headache	Gender	Gender Female	57.8(20.6-162.3)	1.228	57.8(20.6-162.2)
Exertional headache	Full database	All Subjects	50.7(18.4-139.6)	1.208	50.7(18.4-139.6)
Facial paralysis	Age group	Age group 18 - 44 years	5.7(4.4-7.3)	2.057	5.7(4.4-7.2)
Facial paralysis	Age group	Age group 45 - 64 years	6.8(5.1-9.1)	2.218	6.8(5.1-9.0)
Facial paralysis	Age group	Age group ≥ 75 years	11.7(6.8-20.3)	2.161	11.6(6.7-20.1)
Facial paralysis	Gender	Gender Male	12.8(9.7-16.9)	3.069	12.7(9.6-16.7)
Facial paralysis	Gender	Gender Female	6.1(5.0-7.5)	2.258	6.1(5.0-7.5)
Facial paralysis	Age group	Age Group unknown	18.3(11.9-28.1)	3.008	18.1(11.8-27.8)
Facial paralysis	Full database	All Subjects	7.6(6.4-8.9)	2.631	7.6(6.4-8.9)
Facial paresis	Age group	Age group 18 - 44 years	5.2(3.1-8.8)	1.311	5.2(3.1-8.8)
Facial paresis	Age group	Age group 45 - 64 years	5.1(2.5-10.2)	0.861	5.1(2.5-10.2)
Facial paresis	Age group	Age group ≥ 75 years	10.4(3.3-32.4 )	0.093	10.4(3.3-32.3)
Facial paresis	Gender	Gender Male	7.9(3.5-17.6)	0.984	7.9(3.5-17.6)
Facial paresis	Gender	Gender Female	4.7(3.0-7.4)	1.407	4.7(3.0-7.4)
Facial paresis	Full database	All Subjects	5.6(3.8-8.2)	1.750	5.6(3.8-8.2)
Facial spasm	Age group	Age group 18 - 44 years	7.2(3.4-15.2)	1.065	7.2(3.4-15.2)
Facial spasm	Gender	Gender Female	6.2(3.1-12.4)	1.068	6.2(3.1-12.4)
Facial spasm	Full database	All Subjects	6.1(3.2-11.8)	1.173	6.1(3.2-11.8)
Febrile convulsion	Age group	Age group 18 - 44 years	7.2(2.7-19.5)	0.343	7.2(2.7-19.5)
Head discomfort	Age group	Age group 18 - 44 years	2.2(1.5-3.2)	0.544	2.2(1.5-3.2)
Head discomfort	Age group	Age group 45 - 64 years	2.7(1.9-3.8)	0.853	2.7(1.9-3.7)
Head discomfort	Gender	Gender Female	2.1(1.6-2.7)	0.643	2.1(1.6-2.7)
Head discomfort	Gender	Gender Male	2.7(1.5-4.7)	0.393	2.7(1.5-4.7)
Head discomfort	Full database	All Subjects	2.4(1.9-3.0)	0.865	2.4(1.9-3.0)
Headache	Age group	Age group 0 - 27 days	43.9(18.9-101.7)	2.151	35.0(17.9-68.6)
Headache	Age group	Age group 28 days to 23 months	207.9(143.8-300.4)	5.244	147.7(113.4-192.4)
Headache	Age group	Age group 2 - 11 years	13.2(8.9-19.6)	2.560	9.4(7.1-12.3)
Headache	Age group	Age group 12 - 17 years	3.6(1.7-8.0)	0.291	3.1(1.7-5.7)
Headache	Age group	Age group 18 - 44 years	9.0(8.7-9.3)	2.650	6.6(6.4-6.8)
Headache	Age group	Age group 45 - 64 years	8.8(8.4-9.1)	2.645	6.6(6.4-6.8)
Headache	Age group	Age group 65 - 74 years	6.9(5.7-8.3)	2.261	5.8(5.0-6.7)
Headache	Age group	Age group ≥ 75 years	4.6(4.0-5.3)	1.882	4.3(3.8-4.8)
Headache	Age group	Age Group unknown	8.5(7.7-9.4)	2.660	7.0(6.4-7.5)
Headache	Gender	Gender Male	9.9(9.3-10.4)	2.890	7.9(7.5-8.2)
Headache	Gender	Gender Female	8.9(8.6-9.1)	2.680	6.6(6.5-6.8)
Headache	Gender	Gender not known	18.0(15.5-20.9)	3.505	13.4(12.0-14.9)
Headache	Full database	All Subjects	10.0(9.7-10.2)	2.871	7.5(7.4-7.7)
Hemiparaesthesia	Age group	Age group 18 - 44 years	17.1(6.2-46.8)	0.853	17.1(6.2-46.8)
Hemiparaesthesia	Gender	Gender Female	18.3(7.5-44.7)	1.287	18.3(7.5-44.7)
Hemiparaesthesia	Full database	All Subjects	21.1(9.4-47.5)	1.660	21.1(9.4-47.5)
Hemiparesis	Age group	Age group ≥ 75 years	4.9(2.7-8.9)	1.081	4.9(2.7-8.8)
Hyperaesthesia	Age group	Age group 18 - 44 years	2.8(1.9-4.2)	0.797	2.8(1.9-4.2)
Hyperaesthesia	Gender	Gender Female	2.7(1.9-3.8)	0.829	2.7(1.9-3.8)
Hyperaesthesia	Full database	All Subjects	2.7(1.9-3.7)	0.890	2.7(1.9-3.7)
Hypersomnia	Age group	Age group 45 - 64 years	3.0(2.0-4.3)	0.897	3.0(2.0-4.3)
Hypersomnia	Gender	Gender Female	1.7(1.3-2.3)	0.283	1.7(1.3-2.3)
Hypersomnia	Full database	All Subjects	1.6(1.2-2.2)	0.260	1.6(1.2-2.2)
Hypoaesthesia	Age group	Age group 18 - 44 years	2.7(2.4-2.9)	1.233	2.6(2.4-2.9)
Hypoaesthesia	Age group	Age group 45 - 64 years	2.8(2.5-3.1)	1.258	2.7(2.4-3.1)
Hypoaesthesia	Age group	Age group 65 - 74 years	2.7(1.6-4.7)	0.459	2.7(1.6-4.60
Hypoaesthesia	Age group	Age Group unknown	2.3(1.6-3.3)	0.601	2.3(1.6-3.2)
Hypoaesthesia	Gender	Gender Male	2.5(2.0-3.1)	0.985	2.5(2.0-3.0)
Hypoaesthesia	Gender	Gender Female	3.0(2.8-3.3)	1.459	3.0(2.8-3.2)
Hypoaesthesia	Gender	Gender not known	6.5(4.0-10.6)	1.704	6.4(4.0-10.3)
Hypoaesthesia	Full database	All Subjects	3.3(3.1-3.5)	1.586	3.2(3.0-3.5)
Hypogeusia	Age group	Age group 18 - 44 years	4.7(1.9-11.4)	0.278	4.7(1.9-11.4)
Hypogeusia	Gender	Gender Male	6.8(2.5-18.1)	0.305	6.8(2.5-18.1)
Hypogeusia	Full database	All Subjects	3.0(1.5-6.0)	0.252	3.0(1.5-6.0)
Hyporesponsive to stimuli	Gender	Gender Female	4.7(2.0-11.4)	0.285	4.7(2.0-11.4)
Hyposmia	Age group	Age group 18 - 44 years	6.0(2.2-16.0)	0.190	6.0(2.2-16.0)
Hyposmia	Age group	Age group 45 - 64 years	8.1(3.6-18.0)	0.996	8.0(3.6-18.0)
Hyposmia	Gender	Gender Male	9.9(3.2-30.7)	0.067	9.8(3.2-30.6)
Hyposmia	Gender	Gender Female	5.7(2.7-12.1)	0.856	5.7(2.7-12.1)
Hyposmia	Full database	All Subjects	6.6(3.5-12.3)	1.342	6.6(3.5-12.3)
Hypotonia	Age group	Age group ≥ 75 years	5.3(2.4-11.8)	0.605	5.3(2.4-11.7)
Ischaemic stroke	Age group	Age group ≥ 75 years	3.6(2.0-6.6)	0.726	3.6(2.0-6.6)
Lethargy	Gender	Not known	17.0(11.2-25.7)	2.976	16.5(11.0-24.8)
Lethargy	Age group	Age group 18 - 44 years	8.0(7.0-9.1)	2.734	7.9(6.9-8.9)
Lethargy	Age group	Age group unknown	9.0(6.6-12.2)	2.545	8.9(6.6-12.0)
Lethargy	Full database	All Subjects	5.9(5.4-6.5)	2.390	5.8(5.3-6.4)
Lethargy	Gender	Gender Female	5.7(5.1-6.4)	2.317	5.6(5.1-6.3)
Lethargy	Age group	Age group 45 - 64 years	5.8(4.9-6.8)	2.223	5.7(4.8-6.8)
Lethargy	Gender	Gender Male	5.9(4.9-7.2)	2.219	5.9(4.8-7.1)
Lethargy	Age group	Age group ≥ 75 years	6.6(4.8-9.1)	2.096	6.5(4.7-8.9)
Loss of consciousness	Age group	Age group ≥ 75 years	1.8(1.2-2.7)	0.113	1.7(1.2-2.6)
Migraine	Age group	Age group 2 - 11 years	32.6(10.3-102.9)	0.507	31.8(10.4-97.3)
Migraine	Age group	Age group 18 - 44 years	4.3(3.8-4.8)	1.887	4.2(3.8-4.7)
Migraine	Age group	Age group 45 - 64 years	4.1(3.5-4.8)	1.748	4.0(3.4-4.8)
Migraine	Age group	Age group 65 - 74 years	5.0(2.1-12.2.)	0.349	5.0(2.1-12.0)
Migraine	Age group	Age group ≥ 75 years	4.7(2.1-10.6)	0.500	4.7(2.1-10.6)
Migraine	Age group	Age Group unknown	3.5(2.5-5.0)	1.203	3.5(2.5-4.9)
Migraine	Gender	Gender Male	7.5(5.8-9.6)	2.420	7.4(5.8-9.5)
Migraine	Gender	Gender Female	4.0(3.7-4.4)	1.845	4.0(3.6-4.4)
Migraine	Gender	Gender not known	8.2(5.1-13.3)	1.976	8.1(5.0-12.9)
Migraine	Full database	All Subjects	5.2(4.8-5.7)	2.223	5.1(4.7-5.6)
Migraine with aura	Age group	Age group 18 - 44 years	7.3(4.6-11.7)	1.872	7.3(4.6-11.6)
Migraine with aura	Age group	Age group 45 - 64 years	6.7(3.2-14.1)	1.007	6.7(3.2-14.1)
Migraine with aura	Gender	Gender Female	6.7(4.5-10.1)	1.945	6.7(4.5-10.1)
Migraine with aura	Full database	All Subjects	8.4(5.7-12.3)	2.279	8.4(5.7-12.3)
Monoparesis	Gender	Gender Female	3.6(1.6-8.1)	0.209	3.6(1.6-8.1)
Neuralgia	Age group	Age group 18 - 44 years	2.9(2.1-4.1)	0.962	2.9(2.1-4.1)
Neuralgia	Age group	Age group 45 - 64 years	1.9(1.2-2.9)	0.203	1.9(1.2-2.9)
Neuralgia	Gender	Gender Male	2.3(1.2-4.4)	0.008	2.3(1.2-4.4)
Neuralgia	Gender	Gender Female	2.1(1.6-2.7)	0.592	2.1(1.6-2.7)
Neuralgia	Full database	All Subjects	2.3(1.8-3.0)	0.799	2.3(1.8-3.0)
Paraesthesia	Age group	Age group 28 days to 23 months	70.1(22.1-222.2)	0.634	68.6(22.2-212.3)
Paraesthesia	Age group	Age group 18 - 44 years	3.6(3.3-3.9)	1.676	3.5(3.2-3.7)
Paraesthesia	Age group	Age group 45 - 64 years	3.9(3.5-4.2)	1.756	3.7(3.4-4.1)
Paraesthesia	Age group	Age group 65 - 74 years	2.4(1.4-4.0)	0.317	2.4(1.4-4.0)
Paraesthesia	Age group	Age group ≥ 75 years	1.7(1.1-2.6)	0.049	1.7(1.1-2.6)
Paraesthesia	Age group	Age group unknown	3.0(2.3-3.9)	1.138	3.0(2.3-3.8)
Paraesthesia	Gender	Gender Male	3.2(2.7-3.7)	1.380	3.1(2.6-3.6)
Paraesthesia	Gender	Gender Female	4.3(4.0-4.5)	1.949	4.1(3.9-4.4)
Paraesthesia	Gender	Gender not known	3.8(2.4-6.1)	1.065	3.8(2.4-6.0)
Paraesthesia	Full database	All Subjects	4.4(4.2-4.7)	2.021	4.3(4.1-4.6)
Paresis	Age group	Age Group unknown	14.2(4.6-44.3)	0.248	14.2(4.6-44.2)
Parosmia	Age group	Age group 18 - 44 years	2.8(1.7-4.7)	0.574	2.8(1.7-4.7)
Parosmia	Age group	Age group 45 - 64 years	2.4(1.4-4.1)	0.350	2.4(1.4-4.1)
Parosmia	Gender	Gender Female	2.5(1.7-3.7)	0.702	2.5(1.7-3.7)
Parosmia	Full database	All Subjects	2.5(1.8-3.6)	0.763	2.5(1.8-3.6)
Petit mal epilepsy	Age group	Age group ≥ 75 years	12.8(4.1-39.9)	0.195	12.7(4.1-39.8)
Poor quality sleep	Age group	Age group 18 - 44 years	2.4(1.5-3.7)	0.462	2.4(1.5-3.7)
Poor quality sleep	Gender	Gender Female	1.5(1.1-2.2)	0.019	1.5(1.1-2.2)
Poor quality sleep	Full database	All Subjects	1.6(1.1-2.2)	0.133	1.6(1.1-2.2)
Presyncope	Age group	Age group 18 - 44 years	9.1(7.8-10.7)	2.869	9.0(7.7-10.5)
Presyncope	Age group	Age group 45 - 64 years	8.3(6.7-10.4)	2.645	8.3(6.7-10.3)
Presyncope	Age group	Age group 65 - 74 years	4.8(1.8-12.9)	0.017	4.8(1.8-12.8)
Presyncope	Age group	Age group ≥ 75 years	2.9(1.6-5.3)	0.450	2.9(1.6-5.3)
Presyncope	Age group	Age group unknown	14.6(9.6-22.2)	2.805	14.4(9.5-21.9)
Presyncope	Gender	Gender Male	9.8(7.6-12.7)	2.788	9.8(7.6-12.6)
Presyncope	Gender	Gender Female	7.3(6.4-8.3)	2.620	7.2(6.3-8.3)
Presyncope	Gender	Gender not known	17.2(7.7-38.5)	1.551	17.1(7.7-38.0)
Presyncope	Full database	All Subjects	8.4(7.4-9.4)	2.846	8.3(7.4-9.3)
Seizure	Age group	Age group ≥ 75 years	1.9(1.2-3.0)	0.162	1.9(1.2-3.0)
Seizure like phenomena	Full database	All Subjects	3.3(1.6-6.9)	0.247	3.3(1.6-6.9)
Sensory disturbance	Age group	Age group 45 - 64 years	2.7(1.8-4.1)	0.708	2.7(1.8-4.1)
Sensory disturbance	Gender	Gender Female	1.7(1.2-2.4)	0.247	1.7(1.2-2.4)
Sensory disturbance	Full database	All Subjects	1.9(1.4-2.5)	0.433	1.9(1.4-2.5)
Sensory loss	Age group	Age group 18 - 44 years	3.5(2.2-5.5)	0.952	3.5(2.2-5.5)
Sensory loss	Gender	Gender Female	2.8(1.8-4.4)	0.683	2.8(1.8-4.4)
Sensory loss	Full database	All Subjects	2.6(1.7-3.9)	0.617	2.6(1.7-3.9)
Sinus headache	Age group	Age group 18 - 44 years	10.9(7.7-15.5)	2.673	10.9(7.7-15.5)
Sinus headache	Age group	Age group 45 - 64 years	6.8(4.4-10.6)	1.863	6.8(4.4-10.6)
Sinus headache	Gender	Gender Male	7.7(3.5-17.3)	0.969	7.7(3.5-17.3)
Sinus headache	Gender	Gender Female	8.1(6.2-10.7)	2.491	8.1(6.2-10.7)
Sinus headache	Full database	All Subjects	9.3(7.1-12.0)	2.708	9.3(7.2-12.0)
Syncope	Age group	Age group 18 - 44 years	1.8(1.5-2.1)	0.579	1.8(1.5-2.1)
Syncope	Age group	Age group 45 - 64 years	1.6(1.3-2.0)	0.345	1.6(1.3-2.0)
Syncope	Age group	Age group 65 - 74 years	2.6(1.4-4.7)	0.289	2.6(1.4-4.6)
Syncope	Age group	Age Group unknown	2.6(1.7-4.0)	0.627	2.6(1.7-3.9)
Syncope	Gender	Gender Male	1.5(1.2-2.0)	0.171	1.5(1.2-2.0)
Syncope	Gender	Gender Female	1.7(1.5-1.9)	0.526	1.6(1.5-1.9)
Syncope	Full database	All Subjects	1.7(1.5-1.9)	0.573	1.7(1.5-1.9)
Taste disorder	Age group	Age group 18 - 44 years	3.7(2.7-5.2)	1.303	3.7(2.7-5.2)
Taste disorder	Age group	Age group 45 - 64 years	3.5(2.4-5.0)	1.162	3.5(2.4-5.0)
Taste disorder	Age group	Age Group unknown	3.9(1.6-9.3)	0.087	3.9(1.6-9.3)
Taste disorder	Gender	Gender Male	3.3(1.9-5.8)	0.667	3.3(1.9-5.8)
Taste disorder	Gender	Gender Female	3.6(2.8-4.6)	1.419	3.6(2.8-4.6)
Taste disorder	Full database	All Subjects	3.8(3.0-4.7)	1.531	3.8(3.0-4.7)
Tension headache	Age group	Age group 18 - 44 years	18.6(14.2-24.3)	3.535	18.5(14.2-24.2)
Tension headache	Age group	Age group 45 - 64 years	13.0(8.6-19.6)	2.692	12.9(8.6-19.6)
Tension headache	Age group	Age group ≥ 75 years	23.3(9.6-56.6)	1.405	23.2(9.6-56.4)
Tension headache	Age group	Age Group Unknown	26.2(13.6-50.5)	2.392	26.1(13.5-50.2)
Tension headache	Gender	Gender Male	18.7(10.6-33.0)	2.510	18.6(10.6-32.9)
Tension headache	Gender	Gender Female	18.7(15.0-23.4)	3.705	18.7(15.0-23.3)
Tension headache	Gender	Gender not known	32.9(12.2-88.7)	1.113	32.8(12.2-87.9)
Tension headache	Full database	All Subjects	21.0(17.1-25.6)	3.912	20.9(17.1-25.5)
Transient global amnesia	Age group	Age group 45 - 64 years	12.7(4.7-34.3)	0.717	12.7(4.7-34.3)
Transient global amnesia	Gender	Gender Female	8.0(3.0-21.5)	0.425	8.0(3.0-21.5)
Transient global amnesia	Full database	All Subjects	5.9(2.2-15.8)	0.193	5.9(2.2-15.8)
Transient ischaemic attack	Age group	Age group ≥ 75 years	2.2(1.2-3.9)	0.130	2.2(1.2-3.8)
Tremor	Age group	Age group 18 - 44 years	1.7(1.5-1.9)	0.538	1.6(1.5-1.8)
Tremor	Age group	Age group 45 - 64 years	1.6(1.4-1.9)	0.447	1.6(1.4-1.9)
Tremor	Age group	Age group ≥ 75 years	1.5(1.1-2.0)	0.097	1.5(1.1-2.0)
Tremor	Gender	Gender Female	1.7(1.6-1.9)	0.656	1.7(1.6-1.9)
Tremor	Full database	All Subjects	1.7(1.6-1.9)	0.660	1.7(1.6-1.9)
Tunnel vision	Age group	Age group 18 - 44 years	8.8(5.1-15.3)	1.856	8.8(5.1-15.3)
Tunnel vision	Gender	Gender Male	13.6(5.6-32.7)	1.129	13.6(5.6-32.7)
Tunnel vision	Gender	Gender Female	9.0(5.1-15.8)	1.818	9.0(5.1-5.8)
Tunnel vision	Full database	All Subjects	9.9(6.1-15.9)	2.197	9.9(6.1-15.9)
Unresponsive to stimuli	Age group	Age group 65 - 74 years	20.1(10.7-37.6)	2.353	19.8(10.7-36.8)
Unresponsive to stimuli	Age group	Age group ≥ 75 years	14.1(10.0-19.9)	2.993	13.9(9.9-19.6)
Unresponsive to stimuli	Gender	Gender Male	4.4(3.1-6.3)	1.498	4.4(3.1-6.3)
Unresponsive to stimuli	Gender	Gender Female	2.2(1.6-3.0)	0.634	2.2(1.6-3.0)
Unresponsive to stimuli	Full database	All Subjects	2.6(2.1-3.3)	1.011	2.6(2.1-3.3)

## Discussion

In this study, we inspected the VigiBase, the global pharmacovigilance database for the neurological adverse events reported for the COVID-19 vaccine. There was a total of 15,638 adverse events reported from BNT162b2 vaccine, 2,751 from AZD1222 vaccine, 1,075 from mRNA-1273 vaccine, eight from Vero vaccine, two from Covaxin, and for 55 AEs vaccine name was not mentioned.

When the disproportionality analysis was done based on the IC_025_ value, it was found that ageusia, allodynia, anaesthesia, anosmia, aura, balance disorders, burning sensation, cervicobrachial syndrome, cluster headache, dizziness, postural dizziness, dysgeusia, exertional headache, facial paralysis, facial paresis, facial spasm, febrile convulsion, head discomfort, headache, hemiparaesthesia, hemiparesis, hyperaesthesis, hypersomnia, hypoaesthesia, hypogeusia, hyperresponsive to stimuli, hyposomnia, ischemic stroke, lethargy, loss of consciousness, migraine, migraine with aura, monoparesis, neuralgia, paraesthesia, paresis, parosmia, petit mal epilepsy, poor sleep quality, presyncope, seizure, sensory disturbance, sensory loss, sinus headache, syncope, taste disorder, tension headache, transient global amnesia, transient ischemic attack, tremor, tunnel vision, and unresponsive to stimuli are the AEs those could be considered to have an association with the vaccine administration.

Frequently observed adverse events following vaccinations were headache, dizziness, paresthesia, hypoesthesia, lethargy, and migraine. Even though the total number of adverse events were high among individuals receiving BNT162b2 mRNA vaccine yet higher number of events in BNT162b2 mRNA vaccine can be explained as this is the first vaccine to receive emergency use authorization from USFDA, large number doses administration during the analysis period and a shorter interval between the two doses [36].

Polack et al. observed that frequently observed systemic adverse events following BNT162b2 mRNA vaccine second dose administration were fatigue (59%), headache (52%) in the younger population as compared to 51% and 39% among the older population, respectively [16]. In our analysis, headache accounted for 45.46% of the total adverse events with the BNT162b2 vaccine.

The fact sheet for healthcare providers administering BNT162b2 vaccine as published by FDA reported incidence of headache as 55.1% in participants of 16 years of age and older after the first dose and (75.5%) in adolescents of 12 through 15 years of age. In patients of age group 18-55 years reported that the prevalence of headache was 41.9% after dose 1 and 51.7% after dose 2 and 25.2% dose 1 and 39.0 % after dose 2 in 56 years of age and older vaccine recipients [37].

Similar adverse events were reported in the mRNA-1273 vaccine group. In clinical studies, adverse events were not presented as neurological per se, however, this analysis primarily focused on neurological manifestations. Headache was observed in 32.7 % of the total patients after the first vaccination and 58.6% after second vaccination and in both the instances the probability of headache was more in the ≥18 to <65 years group (first vaccination [35.3%] and second vaccination [62.8%]) as compared to the ≥65 years group recipients (first vaccination [24.5%] and second vaccination [46.2%]), respectively [38,39]. Similar results were published by the UK Department of Health and Social Care and the Medicines & Healthcare products Regulatory Agency for the AZD1222 vaccine where reporting AEs related to the nervous system disorders they showed headache as a very common category (Frequencies of occurrence: ≥1/10) which was reported by 52.6% of the vaccine recipients and as followed by dizziness, which was categorized as uncommon (frequencies of occurrence ≥1/1,000 to <1/100) [40]. Bell’s palsy was reported by four participants administered the BNT162b2 and by three participants in the mRNA-1273 vaccine group [37,39]. However, due to insufficient evidence and information, the causal relationship with the vaccines could not be established. The AEs reported with these COVID-19 vaccines may not be a true representation because neurological AEs like headaches are a common occurrence with vaccinations. A study by Cocores et al. conducted on Vaccine Adverse Events Reporting System (VAERS) reported that headache was the fifth most commonly reported adverse event post-vaccination after administration of any vaccine [41].

The disproportionality analysis of this study shows that the reactogenicity of the vaccine among individuals between 18 and 65 years was higher than the individuals above 65 years. This finding was supported by a few previously published clinical studies where a similar type of difference was observed with mRNA-1273, BNT162b2, and AZD1222 vaccines [16,38,42].

Early analysis of all adverse events following vaccination as reported in the COVID-19 vaccine safety update Advisory Committee on Immunization Practices (ACIP) by March 1, 2021, shows that more than 90% were non-serious in nature and headache was the commonest reported AE in both the mRNA vaccines with 20% with BNT162b2 and 23.4 % with the mRNA-1273 vaccine [43]. In a later report as of June 23, 2021, the ACIP in their early safety data of Pfizer-BioNTech vaccination in persons aged 12-15 years and 16-25 years old reported headache as one of the commonest reported adverse events [44]. Similar reports of headache (22.4%) were also reported by the Morbidity and Mortality Weekly Report by CDC, as per the data collected in VAERS in the early phase of vaccination [45]. This difference may be because of the inclusion of local and systemic adverse events, whereas in this study we have included only systemic neurological manifestations. The serious adverse events reported in the VigiBase need to be scrutinized for their association with vaccine administration. In the earlier clinical trials, the majority of serious adverse events were either unrelated to the vaccine or were coincidental due to age-related risk factors. 

Immune reactogenicity to vaccines refers to a group of reactions that are observed immediately after vaccination and are a physical presentation of the inflammatory response to the vaccination. Reactogenicity is determined by either host factors such as age, gender, preexisting immunity or vaccine characteristics, or both [46]. Systemic manifestations of reactogenicity are either due to the vaccine or its adjuvants or both. Presentation of these manifestations varies from fever, headache, dizziness, to seizures and life-threatening anaphylaxis. The systemic pyrogens and other immune mediators cross-talk with the nervous system through the vagus nerve, at the blood-brain barrier, and circumventricular organs. All adjuvant systems induce transient systemic innate responses, which include an increase in the levels of IL-6 and C-reactive protein (CRP), usually peaking at 24 hours after vaccination and subsides to the baseline values within one to three days [47].

The vaccine adjunct can also have a role in reactogenicity. The mRNA vaccines enveloped with lipid nanoparticle (LNP) based delivery system mimics as virus and prevent them from enzymatic digestion along with enhancing immunogenicity without integrating with the genome. However, mRNA itself causes immunogenicity with activation of pro-inflammatory cytokines such as tumor necrosis factor-α (TNF-α), Interleukin (IL)-6 and IL-12 and other innate reactivity manifests as systemic reactions including neurological adverse events. LNP used as an adjuvant precipitates reactogenicity through B-cell amplification and antigen-specific CD4+ and CD8+ T cell responses [48].

Strength of study

This study is based on VigiBase, a large global database of spontaneous reports of AEs used by Uppsala Monitoring Centre, Sweden to generate AEs related signals and used in various published studies to link adverse events with drug use. To avoid any kind of false-positive association, the most conservative method of disproportionality analysis, i.e. IC_025_ values, was used.

Limitations of the study

The data in this study were taken from VigiBase which includes information from varied sources. The probability that the suspected adverse effect is caused by the drug cannot be ascertained for all the cases. The information provided does not represent the opinion of the UMC or the WHO. The data in the study are from a limited duration and to draw conclusive evidence, larger studies with long-term data need to be analyzed.

## Conclusions

Future prospects and conclusion

With numerous molecules being experimented with for the therapy of COVID-19, vaccines have given a ray of hope to curb this pandemic. Almost 15 approved vaccines around the world were being administered in different countries after receiving emergency approval from their respective licensing authorities. These vaccines, in view of an emergency need across the globe, were developed in a short period of time and tried in the clinical trials for a brief period as compared to usual vaccine development. The adverse events with these vaccines are known on the basis of the clinical trials done but long-term and rare adverse events were unclear and are now being revealed with the wide administration drives of vaccination across the globe. All the adverse events associated with any drug or therapy are ultimately reported to the international database called VigiBase hence it is better to analyze their data to find out the common adverse events associated with any therapy across the world. The neurological adverse events associated with these vaccines were not frequent and a headache was one of the common adverse events reported. There were some reported rare serious neurological adverse events but their causal association with the vaccines was to be established. As there can be many predisposing conditions in the patients and interaction with them could also lead to a particular adverse event, hence it was too early to associate a particular adverse event with the vaccines. These reported adverse events can serve as a preliminary signal for potential ADRs associated with these vaccines but based on these results one must not conclude the safety of the above-analyzed vaccines. Moreover, these reported events are adverse events and do not have adverse drug reactions hence definitive causality of the reported events could not be assessed. Therefore long-term robust follow-up studies or cohort studies must be conducted with close monitoring of the adverse events reported establishing the safety of these vaccines.

The current study tries to summarize various neurological manifestations reported in patients who received the vaccines by analyzing the VigiBase, which can help find the common neurological AEs, which can help the prescribers in better management of the public vaccination program and also help in building public awareness and alleviating existing public perceptions.

## Appendices

**Table 2:** Supplementary Table 1: Neurological adverse drug events suspected to be caused by Pfizer vaccine use in COVID-19

Broad heading	Specific adverse event	Non-serious n (%) (n=11,625)	Serious n (%) (n=3,991)	Total n (%) (n=15,616)
Nervous System Disorder(N=15616)	Acoustic neuritis	1(0.01)	0(0.00)	1(0.01)
	Acute disseminated encephalomyelitis	0(0.00)	2(0.05)	1(0.01)
	Ageusia	106(0.91)	32(0.80)	138(0.88)
	Akathisia	1(0.01)	0(0.00)	1(0.01)
	Allodynia	3(0.03)	1(0.03)	4(0.03)
	Altered state of consciousness	1(0.01)	5(0.13)	6(0.04)
	Amnesia	5(0.04)	3(0.08)	8(0.05)
	Anaesthesia	8(0.07)	0(0.00)	8(0.05)
	Anosmia	89(0.77)	25(0.63)	114(0.73)
	Anticholinergic syndrome	0(0.00)	1(0.03)	1(0.01)
	Aphasia	6(0.05)	8(0.20)	14(0.09)
	Areflexia	0(0.00)	4(0.10)	4(0.03)
	Ataxia	2(0.02)	2(0.05)	4(0.03)
	Aura	6(0.05)	2(0.05)	890.05)
	Balance disorder	42(0.36)	25(0.63)	67(0.43)
	Band sensation	3(0.03)	0(0.00)	3(0.02)
	Basal ganglia haemorrhage	0(0.00)	1(0.03)	1(0.01)
	Basilar artery occlusion	0(0.00)	1(0.03)	1(0.01)
	Bradykinesia	2(0.02)	0(0.00)	1(0.01)
	Brain oedema	1(0.01)	0(0.00)	1(0.01)
	Brain stem infarction	0(0.00)	1(0.03)	1(0.01)
	Burning sensation	104(0.89)	30(0.75)	134(0.86)
	Burning sensation mucosal	1(0.01)	0(0.00)	1(0.01)
	Carpal tunnel syndrome	1(0.01)	0(0.00)	1(0.01)
	Central nervous system lesion	0(0.00)	4(0.10)	4(0.03)
	Cerebellar haemorrhage	0(0.00)	1(0.03)	1(0.01)
	Cerebellar infarction	0(0.00)	1(0.03)	1(0.01)
	Cerebellar stroke	0(0.00)	1(0.03)	1(0.01)
	Cerebral artery occlusion	0(0.00)	1(0.03)	1(0.01)
	Cerebral haemorrhage	0(0.00)	6(0.15)	6(0.04)
	Cerebral infarction	0(0.00)	4(0.10)	4(0.03)
	Cerebral venous sinus thrombosis	0(0.00)	1(0.03)	1(0.01)
	Cerebrovascular accident	3(0.03)	22(0.55)	25(0.16)
	Cervicobrachial syndrome	4(0.03)	1(0.03)	5(0.03)
	Cervicogenic headache	1(0.01)	0(0.00)	1(0.01)
	Cholinergic syndrome	1(0.01)	0(0.00)	1(0.01)
	Clonic convulsion	1(0.01)	0(0.00)	1(0.01)
	Clumsiness	1(0.01)	1(0.03)	1(0.01)
	Cluster headache	12(0.10)	6(0.15)	18(0.12)
	Cognitive disorder	1(0.01)	4(0.10)	5(0.03)
	Cold-stimulus headache	1(0.01)	0(0.00)	1(0.01)
	Coma	0(0.00)	2(0.05)	1(0.01)
	Complex regional pain syndrome	1(0.01)	2(0.05)	3(0.02)
	Consciousness fluctuating	0(0.00)	2(0.05)	1(0.01)
	Coordination abnormal	6(0.05)	5(0.13)	11(0.07)
	Cranial nerve disorder	2(0.02)	2(0.05)	4(0.03)
	Dementia	1(0.01)	2(0.05)	3(0.02)
	Dementia of the Alzheimer's type, with delirium	0(0.00)	1(0.03)	1(0.01)
	Demyelination	0(0.00)	1(0.03)	1(0.01)
	Depressed level of consciousness	10(0.09)	11(0.28)	21(0.13)
	Disturbance in attention	52(0.45)	18(0.45)	70(0.45)
	Dizziness	1954(16.81)	595(14.95)	2549
	Dizziness exertional	1(0.01)	1(0.03)	1(0.01)
	Dizziness postural	50(0.43)	29(0.73)	79(0.51)
	Dreamy state	0(0.00)	1(0.03)	1(0.01)
	Drooling	0(0.00)	1(0.03)	1(0.01)
	Dysaesthesia	10(0.09)	1(0.03)	11(0.07)
	Dysarthria	11(0.09)	14(0.35)	25(0.16)
	Dysgeusia	348(2.99)	41(1.03)	389(2.49)
	Dyskinesia	11(0.09)	4(0.10)	15(0.10)
	Dysstasia	19(0.16)	6(0.15)	25(0.16)
	Dystonia	0(0.00)	2(0.05)	1(0.01)
	Encephalitis post immunisation	0(0.00)	1(0.03)	1(0.01)
	Encephalopathy	0(0.00)	1(0.03)	1(0.01)
	Epilepsy	2(0.02)	9(0.23)	11(0.07)
	Essential tremor	1(0.01)	0(0.00)	1(0.01)
	Exertional headache	2(0.02)	1(0.03)	3(0.02)
	Extrapyramidal disorder	0(0.00)	2(0.05)	1(0.01)
	Facial nerve disorder	4(0.03)	0(0.00)	4(0.03)
	Facial neuralgia	2(0.02)	0(0.00)	1(0.01)
	Facial paralysis	799).68)	51(1.28)	130(0.83)
	Facial paresis	9(0.08)	13(0.33)	22(0.14)
	Facial spasm	5(0.04)	2(0.05)	7(0.04)
	Febrile convulsion	1(0.01)	3(0.08)	4(0.03)
	Fine motor skill dysfunction	1(0.01)	1(0.03)	1(0.01)
	Formication	7(0.06)	2(0.05)	9(0.06)
	Generalised tonic-clonic seizure	2(0.02)	7(0.18)	9(0.06)
	Guillain-Barre syndrome	1(0.01)	3(0.08)	4(0.03)
	Haemorrhage intracranial	0(0.00)	5(0.13)	5(0.03)
	Head discomfort	40(0.34)	12(0.30)	52(0.33)
	Head titubation	1(0.01)	0(0.00)	1(0.01)
	Headache	5285(45.46)	1582(39.64)	6867(43.97)
	Hemianaesthesia	3(0.03)	0(0.00)	3(0.02)
	Hemidysaesthesia	1(0.01)	0(0.00)	1(0.01)
	Hemihyperaesthesia	1(0.01)	0(0.00)	1(0.01)
	Hemiparaesthesia	3(0.03)	2(0.05)	6(0.04)
	Hemiparesis	1(0.01)	16(0.40)	17(0.11)
	Horner's syndrome	0(0.00)	1(0.03)	1(0.01)
	Hyperaesthesia	16(0.14)	5(0.13)	21(0.13)
	Hypersomnia	32(0.28)	6(0.15)	389(0.24)
	Hypertonia	2(0.02)	0(0.00)	1(0.01)
	Hypoaesthesia	572(4.92)	144(3.61)	716(4.59)
	Hypogeusia	7(0.06)	0(0.00)	7(0.04)
	Hypokinesia	3(0.03)	3(0.08)	6(0.04)
	Hyporeflexia	1(0.01)	3(0.08)	4(0.03)
	Hyporesponsive to stimuli	2(0.02)	3(0.08)	5(0.03)
	Hyposmia	7(0.06)	2(0.05)	9(0.06)
	Hypotonia	10(0.09)	3(0.08)	13(0.08)
	Incoherent	1(0.01)	0(0.00)	1(0.01)
	Intracranial aneurysm	0(0.00)	1(0.03)	1(0.01)
	Ischaemic stroke	0(0.00)	16(0.40)	16(0.10)
	IVth nerve paralysis	0(0.00)	1(0.03)	1(0.01)
	Lacunar infarction	0(0.00)	2(0.05)	1(0.01)
	Lacunar stroke	0(0.00)	2(0.05)	1(0.01)
	Language disorder	2(0.02)	3(0.08)	5(0.03)
	Lethargy	212(1.82)	139(3.48)	351(2.25)
	Locked-in syndrome	0(0.00)	1(0.03)	1(0.01)
	Loss of consciousness	46(0.40)	40(1.00)	86(0.55)
	Loss of proprioception	0(0.00)	1(0.03)	1(0.01)
	Memory impairment	14(0.12)	7(0.18)	21(0.13)
	Mental impairment	7(0.06)	1(0.03)	8(0.05)
	Migraine	255(2.19)	121(3.03)	376(2.41)
	Migraine with aura	17(0.15)	5(0.13)	22(0.14)
	Migraine without aura	1(0.01)	0(0.00)	1(0.01)
	Mononeuropathy	0(0.00)	1(0.03)	1(0.01)
	Monoparesis	2(0.02)	3(0.08)	5(0.03)
	Monoplegia	3(0.03)	1(0.03)	4(0.03)
	Motor dysfunction	2(0.02)	0(0.00)	1(0.01)
	Movement disorder	13(0.11)	2(0.05)	15(0.10)
	Multiple sclerosis	0(0.00)	1(0.03)	1(0.01)
	Multiple sclerosis relapse	1(0.01)	1(0.03)	1(0.01)
	Muscle contractions involuntary	7(0.06)	2(0.05)	9(0.06)
	Muscle spasticity	0(0.00)	1(0.03)	1(0.01)
	Muscle tone disorder	0(0.00)	1(0.03)	1(0.01)
	Myasthenia gravis	0(0.00)	2(0.05)	1(0.01)
	Myelitis transverse	0(0.00)	1(0.03)	1(0.01)
	Myoclonus	2(0.02)	2(0.05)	4(0.03)
	Narcolepsy	0(0.00)	1(0.03)	1(0.01)
	Nerve compression	3(0.03)	0(0.00)	3(0.02)
	Nervous system disorder	5(0.04)	0(0.00)	5(0.03)
	Neuralgia	37(0.32)	12(0.30)	49(0.31)
	Neuralgic amyotrophy	0(0.00)	3(0.08)	3(0.02)
	Neuritis	2(0.02)	0(0.00)	1(0.01)
	Neurological symptom	3(0.03)	3(0.08)	7(0.04)
	Neuropathy peripheral	12(0.10)	5(0.13)	17(0.11)
	New daily persistent headache	1(0.01)	0(0.00)	1(0.01)
	Noninfective encephalitis	0(0.00)	1(0.03)	1(0.01)
	Nystagmus	2(0.02)	3(0.08)	5(0.03)
	Occipital neuralgia	1(0.01)	0(0.00)	1(0.01)
	Optic neuritis	1(0.01)	1(0.03)	1(0.01)
	Orthostatic intolerance	1(0.01)	0(0.00)	1(0.01)
	Paraesthesia	901(7.75)	201(5.04)	1102(7.06)
	Paralysis	8(0.07)	10(0.25)	18(0.12)
	Paraparesis	0(0.00)	1(0.03)	1(0.01)
	Paresis	5(0.04)	2(0.05)	7(0.04)
	Paresis cranial nerve	1(0.01)	0(0.00)	1(0.01)
	Parkinsonian gait	1(0.01)	0(0.00)	1(0.01)
	Parkinsonism	0(0.00)	1(0.03)	1(0.01)
	Parosmia	24(0.21)	4(0.10)	28(0.18)
	Partial seizures	1(0.01)	0(0.00)	1(0.01)
	Peripheral sensory neuropathy	0(0.00)	3(0.08)	3(0.02)
	Peroneal nerve palsy	0(0.00)	1(0.03)	1(0.01)
	Persistent postural-perceptual dizziness	1(0.01)	0(0.00)	1(0.01)
	Petit mal epilepsy	3(0.03)	1(0.03)	4(0.03)
	Polyneuropathy	1(0.01)	0(0.00)	1(0.01)
	Poor quality sleep	22(0.19)	4(0.10)	26(0.17)
	Posterior reversible encephalopathy syndrome	0(0.00)	1(0.03)	1(0.01)
	Postictal state	2(0.02)	0(0.00)	1(0.01)
	Presyncope	177(1.52)	79(1.98)	256(1.64)
	Primary headache associated with sexual activity	1(0.01)	0(0.00)	1(0.01)
	Psychogenic seizure	1(0.01)	1(0.03)	1(0.01)
	Psychomotor hyperactivity	3(0.03)	1(0.03)	5(0.03)
	Radial nerve palsy	0(0.00)	1(0.03)	1(0.01)
	Radiculitis brachial	0(0.00)	1(0.03)	1(0.01)
	Radiculopathy	2(0.02)	1(0.03)	3(0.02)
	Reduced facial expression	0(0.00)	1(0.03)	1(0.01)
	Restless legs syndrome	7(0.06)	3(0.08)	10(0.06)
	Retinal migraine	0(0.00)	1(0.03)	1(0.01)
	Reversible cerebral vasoconstriction syndrome	0(0.00)	1(0.03)	1(0.01)
	Sciatica	7(0.06)	2(0.05)	9(0.06)
	Sedation	1(0.01)	1(0.03)	1(0.01)
	Seizure	16(0.14)	40(1.00)	56(0.36)
	Seizure like phenomena	2(0.02)	3(0.08)	5(0.03)
	Sensory disturbance	30(0.26)	7(0.18)	37(0.24)
	Sensory loss	10(0.09)	9(0.23)	19(0.12)
	Serotonin syndrome	1(0.01)	0(0.00)	1(0.01)
	Sinus headache	33(0.28)	14(0.35)	47(0.30)
	Slow response to stimuli	2(0.02)	0(0.00)	1(0.01)
	Slow speech	3(0.03)	0(0.00)	3(0.02)
	Small fibre neuropathy	0(0.00)	1(0.03)	1(0.01)
	Somnolence	167(1.44)	49(1.23)	216(1.38)
	Speech disorder	17(0.15)	18(0.45)	35(0.22)
	Status epilepticus	0(0.00)	2(0.05)	1(0.01)
	Stiff leg syndrome	0(0.00)	1(0.03)	1(0.01)
	Stupor	1(0.01)	1(0.03)	1(0.01)
	Subarachnoid haemorrhage	2(0.02)	1(0.03)	3(0.02)
	Sudden onset of sleep	1(0.01)	0(0.00)	1(0.01)
	Syncope	140(1.20)	99(2.48)	239(1.53)
	Taste disorder	50(0.43)	11(0.28)	61(0.39)
	Tension headache	45(0.39)	18(0.45)	63(0.40)
	Thunderclap headache	0(0.00)	1(0.03)	1(0.01)
	Tongue biting	1(0.01)	0(0.00)	1(0.01)
	Tonic clonic movements	2(0.02)	0(0.00)	1(0.01)
	Tonic convulsion	1(0.01)	1(0.03)	1(0.01)
	Transient global amnesia	0(0.00)	3(0.08)	3(0.02)
	Transient ischaemic attack	3(0.03)	9(0.23)	12(0.08)
	Tremor	257(2.21)	128(3.21)	385(2.47)
	Trigeminal neuralgia	5(0.04)	1(0.03)	6(0.04)
	Trigeminal neuritis	1(0.01)	0(0.00)	1(0.01)
	Tunnel vision	10(0.09)	3(0.08)	13(0.08)
	Typical aura without headache	1(0.01)	0(0.00)	1(0.01)
	Unresponsive to stimuli	10(0.09)	37(0.93)	47(0.30)
	Vestibular migraine	0(0.00)	11(0.03)	1(0.01)
	Visual field defect	3(0.03)	3(0.08)	6(0.04)
	Writer's cramp	0(0.00)	1(0.03)	1(0.01)
Investigations (N=22)	Angiogram cerebral abnormal	0(0.00)	1(5.88)	1(4.55)
	Brain natriuretic peptide increased	0(0.00)	2(0.05)	2(9.09)
	Carotid bruit	0(0.00)	1(5.88)	1(4.55)
	Computerised tomogram head abnormal	2(40.00)	0(0.00)	2(9.09)
	CSF protein increased	0(0.00)	2(11.76)	2(9.09)
	Electroencephalogram abnormal	0(0.00)	1(5.88)	1(4.55)
	Magnetic resonance imaging brain abnormal	1(20.00)	6(35.29)	7(31.82)
	Magnetic resonance imaging spinal abnormal	0(0.00)	1(5.88)	1(4.55)
	Neurological examination abnormal	1(20.00)	2(11.76)	3(13.64)
	NIH stroke scale abnormal	0(0.00)	1(5.88)	1(4.55)
	Sensory level abnormal	1(20.00)	0(0.00)	1(4.55)

**Table 3:** Supplementary Table 2: Neurological adverse drug events suspected to be caused by Astra Zeneca vaccine use in COVID-19

Broad heading	Specific adverse event	Non-serious n(%) (n=1,527)	Serious n(%) (n=1,224)	Total n(%) (n=2,751)
Nervous System Disorder(N=2751)	Ageusia	5(0.33)	5(0.41)	10(0.36)
	Allodynia	2(0.13)	1(0.08)	3(0.11)
	Amnesia	1(0.07)	0(0.00)	1(0.04)
	Anosmia	1(0.07)	4(0.330	5(0.18)
	Aphasia	0(0.00)	2(0.16)	2(0.07)
	Balance disorder	2(0.13)	7(0.57)	9(0.33)
	Brain stem stroke	0(0.00)	1(0.08)	1(0.04)
	Burning sensation	0(0.00)	2(0.16)	2(0.07)
	Cerebrovascular accident	0(0.00)	3(0.25)	3(0.11)
	Cervicobrachial syndrome	1(0.07)	0(0.00)	1(0.04)
	Clumsiness	0(0.00)	1(0.08)	1(0.04)
	Cluster headache	4(0.26)	5(0.41)	9(0.33)
	Cognitive disorder	1(0.07)	1(0.08)	2(0.07)
	Coordination abnormal	1(0.07)	0(0.00)	1(0.04)
	Disturbance in attention	2(0.13)	4(0.33)	6(0.22)
	Dizziness	180(11.79)	154(12.58)	334(12.14)
	Dizziness postural	11(0.72)	1290.98)	23(0.84)
	Dysarthria	0(0.00)	1(0.08)	1(0.04)
	Dysgeusia	23(1.51)	11(0.90)	34(1.24)
	Dysstasia	0(0.00)	2(0.16)	2(0.07)
	Epilepsy	0(0.00)	2(0.16)	2(0.07)
	Facial paralysis	2(0.13)	1(0.08)	3(0.11)

**Table 4:** Supplementary Table 3: Neurological adverse drug events suspected to be caused by Moderna vaccine use in COVID-19

Broad heading	Specific adverse event	Non-serious n(%) (n=803)	Non-serious n(%) (n=269)	Total n(%) (n=1,072)
Nervous System Disorder(N=1072)	Ageusia	4(0.50)	0(0.00)	4(0.37)
	Akathisia	1(0.12)	0(0.00)	1(0.09)
	Anosmia	3(0.37)	0(0.00)	3(0.28)
	Anterograde amnesia	0(0.00)	1(0.37)	11(0.09)
	Aphasia	1(0.12)	4(1.49)	5(0.47)
	Aura	1(0.12)	0(0.00)	1(0.09)
	Balance disorder	6(0.75)	1(0.37)	7(0.65)
	Burning sensation	6(0.75)	4(1.49)	10(0.93)
	Cerebral haemorrhage	1(0.12)	0(0.00)	1(0.09)
	Cerebral ischaemia	1(0.12)	0(0.00)	1(0.09)
	Cerebrovascular accident	0(0.00)	4(1.49)	4(0.37)
	Cognitive disorder	0(0.00)	1(0.37)	1(0.09)
	Consciousness fluctuating	0(0.00)	1(0.37)	1(0.09)
	Dementia	0(0.00)	1(0.37)	1(0.09)
	Depressed level of consciousness	0(0.00)	2(0.74)	2(0.19)
	Disturbance in attention	2(0.25)	0(0.00)	2(0.19)
	Dizziness/Dizziness postural	179(22.29)	39(14.50)	218(20.34)
	Drooling	0(0.00)	1(0.37)	1(0.09)
	Dysarthria	4(0.50)	7(2.60)	11(1.03)
	Dysgeusia	20(2.49)	0(0.00)	20(1.87)
	Dysstasia	4(0.50)	1(0.37)	5(0.47)
	Dystonic tremor	0(0.00)	1(0.37)	1(0.09)
	Encephalomalacia	1(0.12)	0(0.00)	1(0.09)
	Facial paralysis	4(0.50)	9(3.35)	13(1.21)
	Facial paresis	0(0.00)	2(0.74)	2(0.19)
	Generalised tonic-clonic seizure	0(0.00)	1(0.37)	1(0.09)
	Guillain-Barre syndrome	0(0.00)	1(0.37)	1(0.09)
	Haemorrhagic stroke	0(0.00)	1(0.37)	1(0.09)
	Head discomfort	5(0.62)	0(0.00)	5(0.47)
	Headache	299(37.34)	37(13.75)	336(31.34)
	Hemianopia	0(0.00)	1(0.37)	1(0.09)
	Hemiparesis	1(0.12)	3(1.12)	4(0.37)
	Hemiplegia	0(0.00)	1(0.37)	1(0.09)
	Hyperaesthesia	1(0.12)	0(0.00)	1(0.09)
	Hypersomnia	7(0.87)	0(0.00)	7(0.65)
	Hypertonia	0(0.00)	1(0.37)	1(0.09)
	Hypoaesthesia	59(7.35)	22(8.18)	81(7.56)
	Hypokinesia	0(0.00)	1(0.37)	1(0.09)
	Hypotonia	0(0.00)	2(0.74)	2(0.19)
	Incoherent/Incoherent Speech disorder	1(0.12)	3(1.12)	4(0.37)
	Intracranial aneurysm	0(0.00)	1(0.37)	1(0.09)
	Ischaemic stroke	0(0.00)	1(0.37)	1(0.09)
	Lacunar infarction	1(0.12)	00(0.00)	1(0.09)
	Lethargy	9(1.12)	3(1.12)	12(1.12)
	Loss of consciousness	9(1.12)	7(2.60)	16(1.49)
	Memory impairment	3(0.37)	6(2.23)	9(0.84)
	Migraine	16(1.99)	3(1.12)	19(1.77)
	Monoplegia	1(0.12)	00(0.00)	1(0.09)
	Motor dysfunction	0(0.00)	1(0.37)	1(0.09)
	Muscle contractions involuntary	1(0.12)	0(0.00)	1(0.09)
	Neuralgia	1(0.12)	1(0.37)	2(0.19)
	Neurologic neglect syndrome	0(0.00)	1(0.37)	1(0.09)
	Neurological symptom	0(0.00)	1(0.37)	1(0.09)
	Opisthotonus	0(0.00)	1(0.37)	1(0.09)
	Paraesthesia	70(8.72)	15(5.58)	85(7.93)
	Poor quality sleep	0(0.00)	1(0.37)	1(0.09)
	Posterior reversible encephalopathy syndrome	0(0.00)	1(0.37)	11(0.09)
	Presyncope	11(1.37)	2(0.74)	13(1.21)
	Reflexes abnormal	0(0.00)	1(0.37)	1(0.09)
	Repetitive speech	0(0.00)	1(0.37)	1(0.09)
	Sedation	1(0.12)	0(0.00)	1(0.09)
	Seizure	0(0.00)	6(2.23)	6(0.56)
	Seizure like phenomena	0(0.00)	1(0.37)	1(0.09)
	Sensory disturbance	3(0.37)	2(0.74)	5(0.47)
	Sinus headache	1(0.12)	0(0.00)	1(0.09)
	Somnolence	8(1.00)	4(1.49)	12(1.12)
	Speech disorder	1(0.12)	11(4.09)	12(1.12)
	Syncope	11(1.37)	11(4.09)	22(2.25)
	Taste disorder	0(0.00)	2(0.74)	2(0.19)
	Thrombotic stroke	0(0.00)	1(0.37)	1(0.09)
	Transient ischaemic attack	0(0.00)	1(0.37)	1(0.09)
	Tremor	38(4.73)	11(0.37)	49(4.57)
	Tunnel vision	3(0.37)	0(0.00)	3(0.28)
	Unresponsive to stimuli	3(0.37)	19(7.06)	22(2.05)
	Visual field defect	1(0.12)	0(0.00)	1(0.09)
Investigations(N=3)	Coma scale abnormal	0(0.00)	1(50.00)	1(33.33)
	Computerised tomogram head abnormal	0(0.00)	1(50.00)	1(33.33)
	Magnetic resonance imaging brain abnormal	1(0.12)	0(0.00)	1(33.33)

**Table 5:** Supplementary Table 4: Neurological adverse drug events suspected to be caused by Vero vaccine use in COVID-19

Broad heading	Specific adverse event	Non-serious n(%) (n=4)	Serious n(%) (n=2)	Total n(%) (n=6)
Nervous System Disorder (N=6)	Cerebral venous sinus thrombosis	0(0.00)	1(50.0)	1(16.77)
	Dizziness	4(100.0)	0(0.00)	4(66.67)
	Headache	0(0.00)	1(50.0)	1(16.67)

**Table 6:** Supplementary Table 5: Neurological adverse drug events suspected to be caused by Covaxin vaccine use in COVID-19

Broad heading	Specific adverse event	Non-serious n(%) (n=2)	Serious n(%) (n=0)	Total n(%) (n=2)
Nervous System Disorder(N=2)	Headache	2(100.0)	0(0.00)	2(100.0)

**Table 7:** Supplementary Table 6: Neurological adverse drug events suspected to be caused by unknown vaccine use in COVID-19

Broad heading	Specific adverse event	Non-serious n (%) (n=39)	Serious n (%) (n=16)	Total n(%) (n=55)
Nervous System Disorder(n=55)	Balance disorder	0(0.00)	1(6.25)	1(1.82)
	Cerebrovascular accident	0(0.00)	1(6.25)	1(1.82)
	Dizziness	5(12.82)	2(12.5)	7(12.73)
	Dysgeusia	1(2.56)	1(6.25)	2(3.64)
	Dysstasia	0(0.00)	1(6.25)	1(1.82)
	Headache	22(56.41)	4(25.0)	26(47.27)
	Hypoaesthesia	1(2.56)	0(0.00)	1(1.82)
	Lethargy	1(2.56)	1(6.25)	2(3.64)
	Migraine	1(2.56)	1(6.25)	2(3.64)
	Paraesthesia	1(2.56)	0(0.00)	3(5.45)
	Presyncope	1(2.56)	1(6.25)	2(3.64)
	Sinus headache	1(2.56)	0(0.00)	1(1.82)
	Syncope	1(2.56)	1(6.25)	2(3.64)
	Taste disorder	1(2.56)	0(0.00)	1(1.82)
	Transient ischaemic attack	0(0.00)	1(6.25)	1(1.82)
	Tremor	1(2.56)	1(6.25)	2(3.64)
